# The ICH score combined with the CRP/albumin ratio as an improved early prognostic tool for in-hospital mortality in spontaneous intracerebral hemorrhage

**DOI:** 10.1016/j.bas.2026.106084

**Published:** 2026-05-04

**Authors:** K. Haferkorn, M. Bender, E. Uhl, M. Stein

**Affiliations:** University hospital Giessen and Marburg, Department of Neurosurgery, Giessen, Germany

**Keywords:** Intracerebral hemorrhage, ICH-Score, CRP/Albumin ratio, In-hospital mortality, Neurocritical care, Risk stratification

## Abstract

**Introduction:**

The ICH score is a widely used tool for predicting mortality in spontaneous intracerebral hemorrhage (sICH) patients, but its accuracy for early in-hospital prognosis remains limited. Recently, the C-reactive protein (CRP)/albumin ratio has been identified as a potential biomarker associated with in-hospital mortality (IHM) in sICH patients.

**Research question:**

This study aimed to investigate whether combining the established ICH score with the CRP/albumin ratio at admission improves the prediction of IHM in sICH patients.

**Material and methods:**

A retrospective analysis was conducted including 379 patients with sICH treated in an intensive care unit between 02/2008 and 12/2017. Based on previous findings identifying a CRP/albumin ratio >1.22 as an independent predictor of IHM, the ICH score was modified by adding one point when this threshold was exceeded. Prognostic performance of the original ICH score and the modified ICH (mICH) score was evaluated using receiver operating characteristic (ROC) analysis in the entire cohort and in subgroups of patients with Glasgow Coma Scale (GCS) ≤ 8 and intraventricular hemorrhage.

**Results:**

The mICH score demonstrated improved prognostic performance compared with the original ICH score in the overall cohort (AUC 0.776 vs. 0.761). In patients with severe neurological impairment (GCS ≤8), the predictive accuracy increased from an AUC of 0.672 to 0.719. Similarly, in patients with intraventricular hemorrhage, the AUC improved from 0.747 to 0.774. The modified score showed higher sensitivity and improved Youden index values across analyses.

**Discussion and conclusion:**

Incorporating the CRP/albumin ratio into the original ICH score was associated with modestly improved discrimination for in-hospital mortality, with more pronounced gains in patients presenting with GCS ≤8 or intraventricular hemorrhage.

## Highlights

• Combining the original ICH score with the CRP/albumin ratio improves prediction of in-hospital mortality.
• The modified ICH score shows higher prognostic accuracy than the original ICH score.
• Prognostic improvement is strongest in patients with initial GCS ≤8.
• The modified score also performs better in patients with intraventricular hemorrhage.

## 1 Introduction

Non-traumatic intracerebral hemorrhage (ICH) is the most common form of stroke leading to death, with a 30-day mortality rate of 40% and a 1-year mortality rate of 50% (Poon et al., 2014; van Asch et al., 2010). Survivors show severe neurological deficits in 61 - 88 % of cases and only 12 - 39 % of those affected are able to resume their previous life (van Asch et al., 2010). The prognosis of the clinical course is difficult to assess in the first few days after the bleeding event. According to current guidelines in patients with acute intracerebral hemorrhage resuscitation measures should not be withheld, treatment not discontinued or the intensity of treatment not be limited within the first 48 h (Greenberg et al., 2022; Steiner, 2021). However, this does not apply to patients with a clear initial unfavorable prognosis and/or the existence of a clear living will. However, there is currently a lack of precise prognostic parameters that can help treating physicians to decide whether and to what extent intensive medical treatment should be started, continued, extended or possibly even discontinued in patients with intracerebral hemorrhage. In addition to established prognostic parameters such as age, hematoma volume, and initial clinical condition—summarized in the ICH score (Hemphill et al., 2001)—serum biomarkers including C-reactive protein (CRP), the neutrophil-to-lymphocyte ratio, and troponin I (TNI) have increasingly attracted research interest in recent years (Bender et al., 2021; Di Napoli et al., 2011; Giede-Jeppe et al., 2017).

The usefulness of the CRP/albumin ratio for predicting intrahospital mortality in patients with intracerebral hemorrhage in the neurosurgical intensive care unit has already been investigated by Bender et al. (2020). In this study, an admission CRP/albumin ratio >1.22 was associated with an increased risk of in-hospital mortality. This study aimed to improve prognostic assessment in patients with ICH by combining the admission CRP/albumin ratio with the ICH score.

## 2 Study design and patients

The collection of patient data has been described previously (Bender et al., 2020). The study design has been approved by the Ethics Committee of the Department of Medicine at Justus Liebig University (AZ 95/17, July 2017; Amendment, February 2019). In summary, this study included all patients with spontaneous ICH treated in the neurosurgical intensive care unit of the University Hospital Giessen and Marburg (Giessen) between February 2008 and December 2017. After the initial examination in the emergency department, a blood sample was obtained that included CRP, albumin, and additional serum biomarkers (e.g., leukocytes, hemoglobin, hematocrit, troponin I, lactate). Cerebral imaging was performed using cranial computed tomography, followed by either immediate surgical intervention or admission to the neurosurgical intensive care unit. Inclusion criteria comprised patients aged >18 years with spontaneous ICH and a minimum stay of 24 h in the neurosurgical intensive care unit. Exclusion criteria were ICH secondary to trauma, cerebral vascular malformations, or cerebral neoplasia, as well as acute or chronic liver failure.

### 2.1 Study parameters

The primary study parameter was the modified intracerebral hemorrhage score. In addition, demographic, laboratory and radiological data, intensive care parameters, the Glasgow Coma Score (GCS) and the modified Rankin Scale (mRS) were determined.

### 2.2 Imaging

The initial cranial computed tomography scan was analyzed according to the hemorrhage location and size as well as occurrence of intraventricular hemorrhage. Hemorrhage localization was differentiated into lobar, deep lying and cerebellar. The hemorrhage size (ml) was calculated using formula A x B x C/2 (Kothari et al., 1996). The variables A, B and C represent the diameters of the three dimensions of the hemorrhage at right angles to each other. The Graeb score (Graeb et al., 1982) was also used for a more precise assessment of intraventricular blood components.

### 2.3 Modified intracerebral hemorrhage score (mICH score)

The ICH score (Hemphill et al., 2001) was determined on the basis of imaging and clinical data. Patients with a CRP/albumin ratio >1.22 received an additional point (modified ICH score, Table 1). This ratio was the cut-off value for a higher risk of intrahospital death in our previous study. For a detailed evaluation, subgroups of patients with a GCS ≤8 and of patients with intraventricular hemorrhage were analyzed.

**Table 1:** Modified Intracerebral Hemorrhage (mICH) Score; GCS – Glasgow Coma Score; CRP – C-reactive protein.

Parameters	mICH-Score points
**GCS-Score**	
3-4	2
5-12	1
13-15	0

**ICH-volume (cm^3^)**	
≥ 30	1
< 30	0

**Intraventricular hemorrhage**	
Yes	1
No	0

**Infratentoriell ICH**	
yes	1
no	0

**Age (years)**	
≥ 80	1
< 80	0

**CRP-/Album-Ratio**	
> 1,22	1
< 1,22	0

**mICH-Score overall**	0-7

### 2.4 Statistics

Baseline data of the patients and the calculation of the cut off value of the CRP/albumin ratio were described in detail previously (Bender et al., 2020). Absolute and relative frequencies were calculated for the descriptive statistics. Normally distributed parameters were presented as arithmetic mean ± standard deviation (SD) and the non-normally distributed parameters as median and interquartile range (IQR). The study population was divided into two groups: Survivors and non-survivors. The calculation was performed using SPSS software (version 15.0; SPSS Inc., Chicago, USA). In order to determine a threshold value at which the distinction between the two analyzed groups (not deceased vs. deceased) will succeed with the greatest possible probability, the Youden Index and the area under the curve were calculated in a receiver operating curve (ROC) analysis using R statistical software (version 3.4.1, RCore Team, 2017; Dormagen, Germany).

An ROC analysis of the ICH score alone compared with the ICH score combined with the CRP/albumin ratio was then performed for all included patients in order to estimate the extent to which the CRP/albumin ratio changes the accuracy of the prognosis (IBM SPSS Statistics 29.0.2.0). Subsequently, two subgroups - patients with a GCS ≤8 and patients with intraventricular hemorrhage - were also subjected to a ROC analysis of the ICH score vs. the mICH score for further evaluation. Furthermore, all groups were subjected to an overall model quality analysis to better assess the predictive quality of the calculations.

## 3 Results

### 3.1 Study population

In the period from February 2008 to December 2017, a total of 759 patients with intracerebral hemorrhage of various origins were treated in our neurosurgical intensive care unit. Taking into account the exclusion criteria, 379 cases with spontaneous intracerebral hemorrhage were included. The mean age was 68.2 ± 13.3 years (range: 18 - 93 years, Table 2) and the proportion of women was 44.9% (n = 170). The overall collective showed a median GCS of 8 on admission (IQR: 3-12).

**Table 2:** Baseline data of the study population. SD: standard deviation, IQR: interquartile range, APACHE II: Acute Physiology and Chronic Health Evaluation II, ICH: intracerebral hemorrhage, IVH: intraventricular hemorrhage, mRS: modified Rankin Scale.

Parameters	Results
**Baseline Data**	
Age, years, Mean (±SD)^a^	68.2 (13.3)
Women, n (%)	170 (44.9)
Men, n (%)	209 (55.1)
Glasgow Coma Scale, Median (IQR)^a^	8 (3-12)
⁃ Glasgow Coma Scale ≤8, n (%)	210 (55.4)

***Imaging***^a^	
Localization	
⁃ Supratentorial, lobar, n (%)	129 (34.0)
⁃ Supratentorial, deep, n (%)	194 (51.2)
⁃ Infratentorial, n (%)	56 (14.8)
ICH Volume, cm^3^, Mean (±SD)	51.8 (42.3)
IVH, n (%)	269 (71.0)

***Biomarkers***	
C-reactive Protein, mg/L, Mean (±SD)^a^	22.1 (38.9)
Albumin, g/L, Mean (±SD)^a^	38.1 (5.6)
C-reactive Protein/Albumin Ratio, Mean (±SD)^a^	0.63 (1.1)

***Clinical Outcome***^b^	
mRS, Median (IQR)	5 (4-6)
⁃ mRS 0, n (%)	0 (0)
⁃ mRS 1, n (%)	21 (5.5)
⁃ mRS 2, n (%)	27 (7.1)
⁃ mRS 3, n (%)	26 (6.9)
⁃ mRS 4, n (%)	77 (20.3)
⁃ mRS 5, n (%)	110 (29.0)
⁃ mRS 6, n (%)	118 (31.1)

a Upon admission.

b At discharge.

### 3.2 Imaging

On cranial computed tomography, 51.2 % of all patients showed a deep supratentorial bleeding localization, 34,0 % a lobar supratentorial bleeding and 14.8 % an infratentorial bleeding (Table 2). The average bleeding volume was 51.8 ± 42.3 cm^3^. Intraventricular hemorrhage was present in 269 patients (71%).

### 3.3 Clinical outcome

During the inpatient stay, 118 patients (31.1 %) died, 45 of whom were women (38.1 %) and 73 men (61.9 %, Table 2). The median mRS score was 5 (IQR 4-6). An unfavorable outcome (mRS 4-6) was present in 305 patients (80.5 %).

### 3.4 ROC analysis of the ICH score compared with the mICH score

**Fig. 1:**
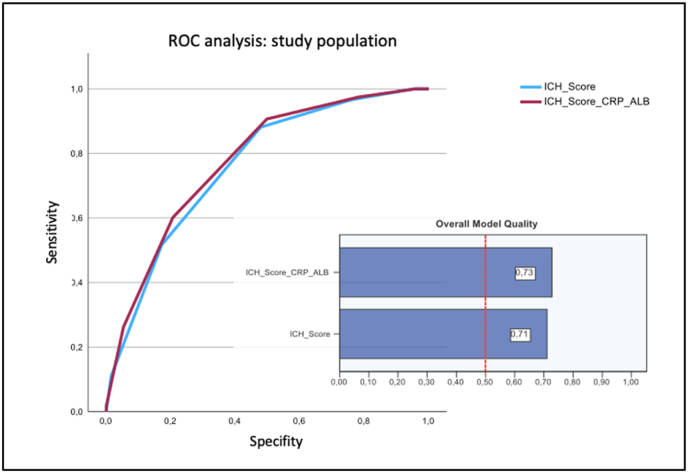
Comparison of ROC analysis of the ICH score alone and in combination with the CRP/albumin ratio of all patients.

The ROC analysis of the ICH score in combination with the CRP/albumin ratio of all patients showed a higher area under the curve (AUC: 0.776, cut-off: ≥3, sensitivity: 0.907, specificity: 0.498, Youden index: 0.409) than the ICH score alone (AUC: 0.761, cut-off: ≥3, sensitivity: 0.881, specificity: 0.479, Youden index: 0.402), with generally good model quality (Fig. 1).

**Fig. 2:**
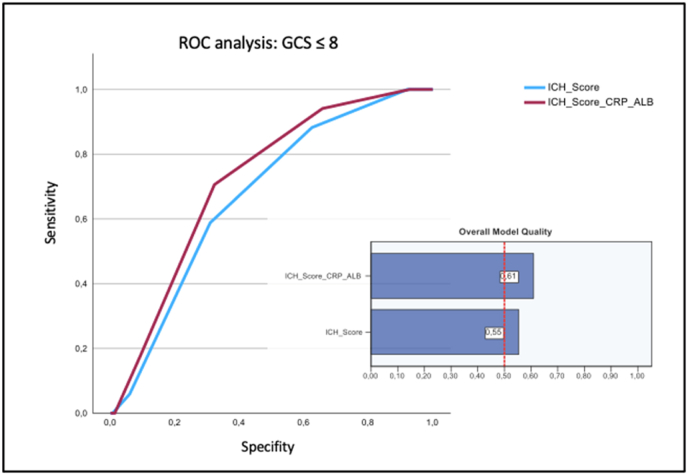
Comparison of ROC analysis of the ICH score alone and in combination with the CRP/albumin ratio of ICH patients with initial GCS ≤8.

For patients with intracerebral hemorrhage and an initial GCS ≤8 (n = 169), there was an improved prediction of intrahospital mortality for the mICH score (AUC: 0.719, cut-off: ≥3, sensitivity: 0.706, specificity: 0.322, Youden index: 0.384) compared with the ICH score alone (AUC: 0.672, cut-off: ≥3, sensitivity: 0.588, specificity: 0.309, Youden index: 0.279), with also generally good model quality (Fig. 2).

**Fig. 3:**
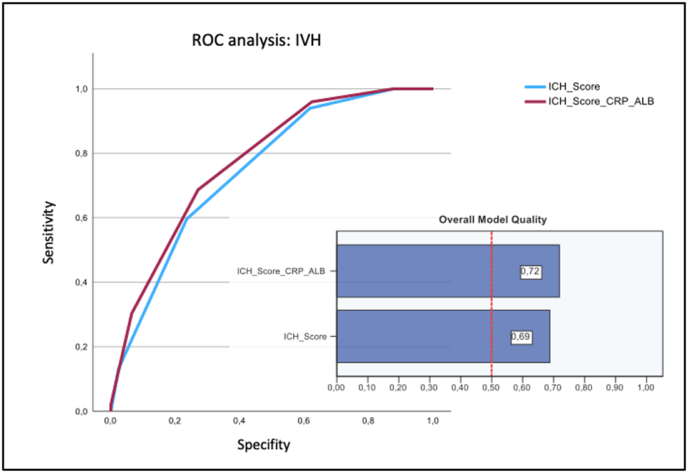
Comparison of ROC analysis of the ICH score alone and in combination with the CRP/albumin ratio of ICH patients with concomitant intraventricular hemorrhage.

For patients with intracerebral and concomitant intraventricular hemorrhage (n = 269), the mICH score also showed improved prognostic features regarding intrahospital mortality (AUC: 0.774, cut-off: ≥4, sensitivity: 0.687, specificity: 0.271, Youden index: 0.416) compared with the ICH score alone (AUC: 0.747, cut-off: ≥4, sensitivity: 0.596, specificity: 0.235, Youden index: 0.361), with repeatedly good model quality (Fig. 3).

## 4 Discussion

The observed improvement in AUC in the overall cohort was numerically small and should therefore be interpreted cautiously. However, consistent increases in sensitivity and Youden index suggest potential utility for early risk enrichment in selected critically ill patients. The mICH score should be considered a complementary bedside tool that may support, but never replace, comprehensive clinical judgment.

Our results support the clinical utility of the mICH score particularly in patients with intraventricular hemorrhage and in those presenting with severe impairment of consciousness (GCS ≤8). In these subgroups, the modified score provides a more reliable estimation of the risk for intrahospital mortality and thus represents a meaningful step toward more precise and individualized prognostication after spontaneous intracerebral hemorrhage.

The best known and easiest to use score for predicting 30-day mortality after spontaneous intracerebral hemorrhage is the ICH score (Hemphill et al., 2001). The five modalities age, GCS, hematoma size and localization as well as the presence of intraventricular hemorrhage can be determined quickly and precisely in the central emergency room and with the help of a cCT.

However, limitations exist, such as the dichotomization of age and the restricted ability to predict very early or intrahospital mortality. As a consequence, several modified ICH scores have been developed, integrating additional clinical parameters or laboratory data, but their general applicability is limited. Nisar et al. were able to show that age >80 years does not significantly contribute to 30-day mortality (Nisar et al., 2018). Various studies changed the age as well as other parameters (e.g. NIHSS instead of GCS, oral anticoagulation, respiratory insufficiency) and developed modified ICH scores, which gave them improved predictive validity for mortality after spontaneous intracerebral hemorrhage (Sembill et al., 2017; Widyadharma et al.). Based on data from the INTERACT II study, Heeley et al. compared the original ICH score (Hemphill et al., 2001) with a modified ICH score (Cheung and Zou), which used the NHISS instead of the GCS, and with the Intracerebral Grading Scale (Ruiz-Sandoval et al.), which includes additional points for different age groups, hemorrhage locations and volumes (Heeley et al.). They concluded that although the prognostic scores offer a good predictive probability for 90-day mortality and severe disability at 90 days, they are not suitable for predicting intrahospital or short-term mortality.

In recent years, a variety of serum biomarkers have been explored as potential predictors of outcome and short-term mortality following spontaneous ICH. Among the most frequently investigated are troponin-I (TNI), C-reactive protein (CRP), D-dimers, blood glucose, leukocyte count, the neutrophil-to-lymphocyte ratio, cortisol, and albumin. However, the clinical significance of these markers remains to be established (Bender et al., 2021; Di Napoli et al., 2011; Giede-Jeppe et al., 2017; Agnihotri et al., 2011; Diedler et al., 2009; Garrett et al., 2010; Gerner et al., 2018; Hu et al., 2014; Wu et al., 2017; Yang et al., 2014; Zheng et al.).

In a previous study, we were able to show that there is a significant correlation between an initially elevated CRP/albumin level and intrahospital mortality in patients with spontaneous intracerebral hemorrhage treated in a neurosurgical intensive care unit. A CRP/albumin ratio >1.22 on admission was an independent predictor of intrahospital mortality in these patients.

The present study demonstrates, for the first time, that the incorporation of the CRP/albumin ratio into the ICH score (mICH score) enhances prognostic accuracy for in-hospital mortality. Although the overall improvement in the entire cohort was modest, the effect was more pronounced in clinically critical subgroups. In patients presenting with a GCS ≤8, the mICH score substantially improved predictive performance compared with the original ICH score (AUC increase from 0.672 to 0.719; Youden index from 0.279 to 0.384). Similarly, in patients with intraventricular hemorrhage, the mICH score outperformed the original ICH score (AUC increase from 0.747 to 0.774; Youden index from 0.361 to 0.416).

In this context, serum biomarkers represent a promising extension of established prognostic scores. Among these, the CRP/albumin ratio is particularly appealing, as it integrates systemic inflammation and nutritional status—two factors independently associated with outcome. Moreover, it can be obtained rapidly and objectively at admission, without reliance on detailed medical history, which is often unavailable in patients with impaired consciousness.

Given that a complete medical history is often unobtainable in the acute setting of ICH due to impaired consciousness, aphasia, or other neurological deficits, rapidly accessible and objective parameters such as serum biomarkers are of increasing relevance for the development of prognostic models. In this context, the ICH-CRP/albumin ratio score appears particularly valuable for estimating in-hospital mortality in critically ill patients presenting with an initial GCS ≤8 or intraventricular hemorrhage.

### 4.1 Limitations

This retrospective single-center analysis is vulnerable to selection bias, unmeasured confounding, and center-specific treatment effects. The CRP/albumin threshold was derived from the same institutional cohort, which may overestimate performance. Calibration and net-benefit analyses were not available. Data were collected between 2008 and 2017 in a single-center setting, which limits generalizability. Nevertheless, the large case volume from a university hospital within a defined timeframe lends robustness to the findings. Finally, only one serum biomarker was incorporated into the ICH score. Future prospective studies are warranted to assess whether additional biomarkers or clinical parameters may further improve the sensitivity and specificity of prognostic models.

## 5 Conclusion

The addition of the CRP/albumin ratio to the ICH score may modestly improve early prediction of in-hospital mortality after spontaneous ICH, particularly in patients with severe presentation. These results are hypothesis-generating and warrant prospective confirmation.

In addition, the combination with other serum biomarkers and other scores is an interesting approach for further prospective studies.

## Ethics Committee

The study design has been approved by the Ethics Committee of the Department of Medicine at Justus Liebig University (AZ 95/17, July 2017; Amendment, February 2019). No human participants included.

## Declaration of competing interest

The authors declare that they have no known competing financial interests or personal relationships that could have appeared to influence the work reported in this paper.
